# Supplementary material for: In Vitro Osteogenesis Study of Shell Nacre Cement with Older and Young Donor Bone Marrow Mesenchymal Stem/Stromal Cells
Source: Bioengineering (Basel). 2024 Jan 31;11(2):143. doi: 10.3390/bioengineering11020143 (PMC10886325; doi:10.3390/bioengineering11020143)
Supplement: Supplementary file 1 [file bioengineering-11-00143-s001.zip › bioengineering-2752967-supplementary.pdf]

Supplementary table S1: List for genes and probes for qPCR

No	Gene Name	Gene ID	Assay	Category
1.	Bone Morphogenetic Protein 2	BMP2	Hs00154192_m1	Osteogenesis
2.	Runt related transcription factor 2	RUNX2	Hs00231692_m1	
3.	Alkaline phosphatase	ALP	Hs00758162_m1	
4.	Collagen Type I Alpha 1 Chain	COL1A1	Hs01076777_m1	
5.	Osteomodulin	OMD	Hs00192325_m1	
6.	Secreted Protein Acidic And Cysteine Rich	SPARC	Hs00277762_m1	
7.	Cyclin dependent kinase inhibitor 2A (AKA p16)	p16/CDKN2A	Hs00923894_m1	Senescence/cell cycle
8.	Cyclin dependent kinase inhibitor 1A (AKA p21)	p21/CDKN1A	Hs00355782_m1	
9.	Tumour protein 53	p53	Hs01034249_m1	
10.	Hypoxanthine phosphoribosyl transferase 1	HPRT1	Hs99999909_m1	House-keeping gene
